# Penetrating skull stab

**DOI:** 10.1007/s12024-024-00926-0

**Published:** 2024-12-05

**Authors:** Claas Buschmann, Johanna Preuß-Wössner, Christoph Meißner

**Affiliations:** 1 Institute of Legal Medicine, University Hospital Schleswig-Holstein, Arnold-Heller-Str. 3, Building 28, 24105 Kiel, Germany; 2 Institute of Legal Medicine, University Hospital Schleswig-Holstein, Kahlhorststr. 31-35, Building 89, 23562 Lübeck, Germany

**Keywords:** Autopsy, Sharp force, Penetrating injury, Neuropathology

## Abstract

Fatal skull stabs are rare. In the case reported here, a 28-year-old man sustained an isolated penetrating skull injury from a knife and died two days later. The bone shard with the stab puncture, which was neurosurgically removed before death, later allowed the reproducible exact assignment of the murder knife found at the scene to the stab as well as the estimation of the length of the intracranial stab channel. This, together with the findings of the formalin-fixed brain, allowed forensic reconstruction.

## Case report

**Fig. 1:**
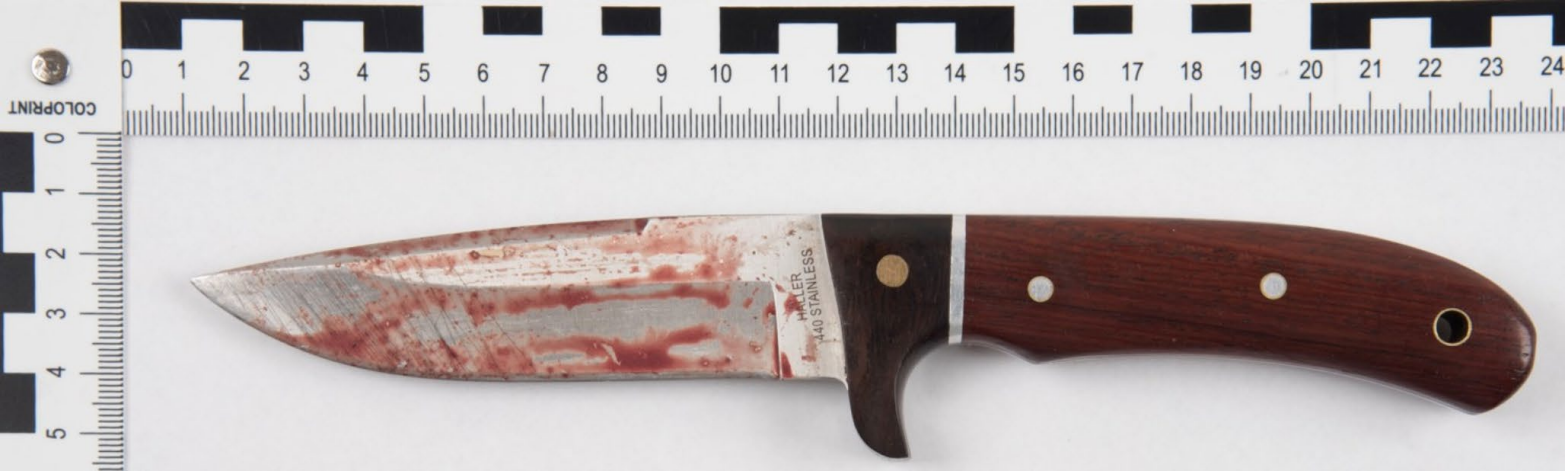
Murder knife

A 28-year-old man sustained an isolated stab to the head and was taken to hospital by emergency service. A cranial computed tomography revealed an approximately 10 cm deep stab wound from the left parietal to the right with bleeding in the stab channel, particularly on the right, a subdural hematoma on the left frontoparietotemporal and in the interhemispheric space, and intraventricular hemorrhage on both sides with tamponade of the ventricles up to the 4th ventricle. The patient underwent immediate neurosurgical intervention. However, he died about two days later from brainstem compression as a result of brain swelling from the stab. The murder knife was recovered on site (Fig. 1). The perpetrator was arrested abroad and later sentenced to 9 years in prison for manslaughter.

### Autopsy

Autopsy revealed the cause of death to be a stab wound to the left upper head and both hemispheres with subsequent herniation of the brainstem in the foramen magnum despite neurosurgical treatment. No further injuries were found. The original stab wound in the scalp could not be defined after neurosurgical intervention as it was included in the surgical incision to remove the bone shard damaged by the stab. The bone shard with a thickness of 0.5 cm showed a stab-induced impression fracture tapering towards the midline of the body through the skull with a corresponding parenchymal wound in the left cerebral hemisphere. There were small fractures next to the penetrating bony injury affecting the tabula externa as well as the tabula interna (Fig. 2a and 2b).

**Fig. 2a:**
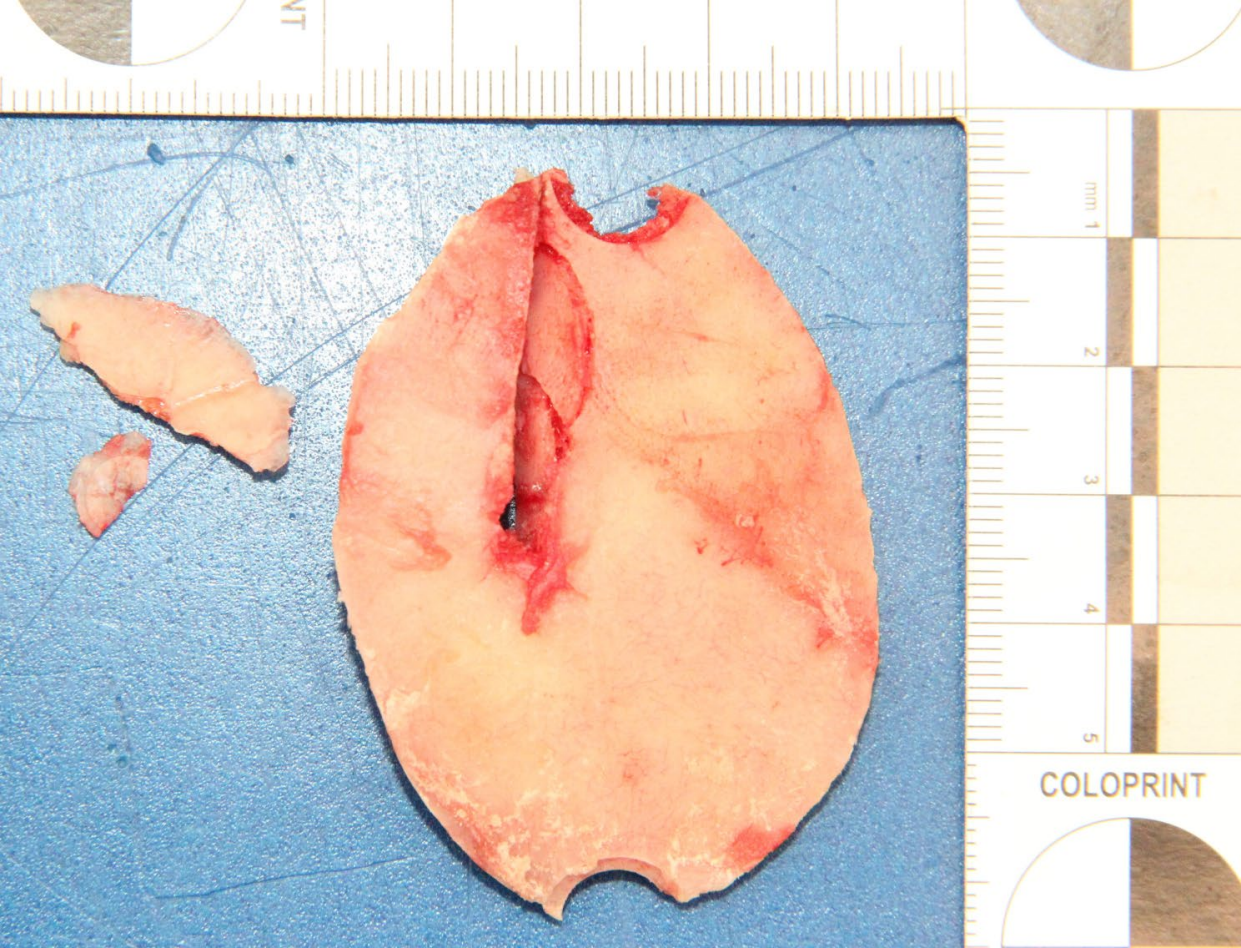
Bone shard with stab-induced impression fracture

**Fig. 2b:**
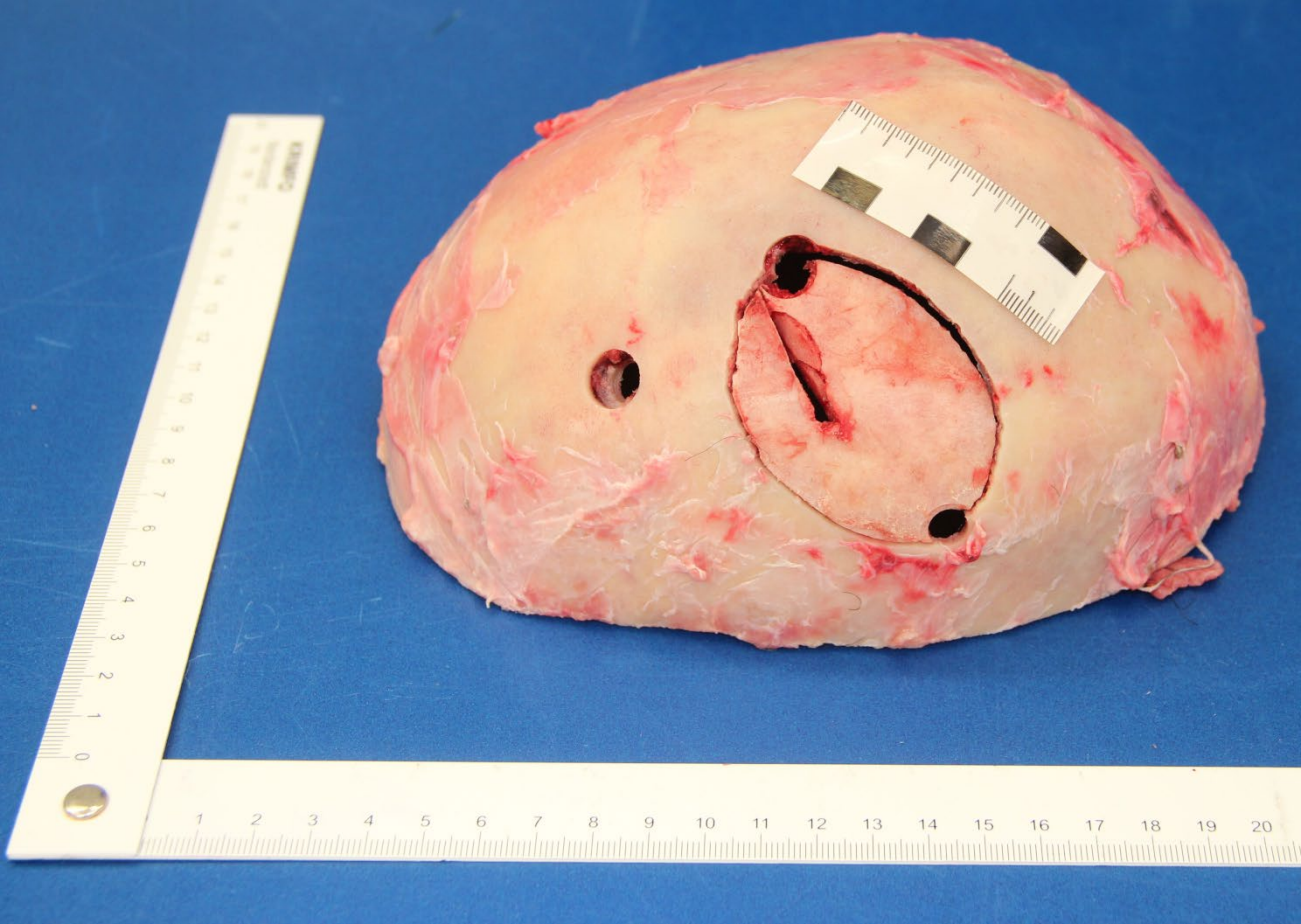
Bone shard and corresponding skull

The brain was subsequently processed after formalin fixation. There was an area approximately measuring 7 cm in the transverse axis and 6.5 cm in the slightly oblique longitudinal axis over the left cerebral hemisphere with subsequent road-like tissue destruction into the right cerebral hemisphere, severe bleeding there, ventricular tamponade, massive cerebral edema and - histologically - pressure-related pontine hemorrhage (Fig. 3a and 3b). The murder weapon and the bone shard corresponded exactly and showed an approximately 10 cm long intracranial stab channel with alteration of intracranial vital structures [Fig. 4a and 4b].

**Fig. 3a:**
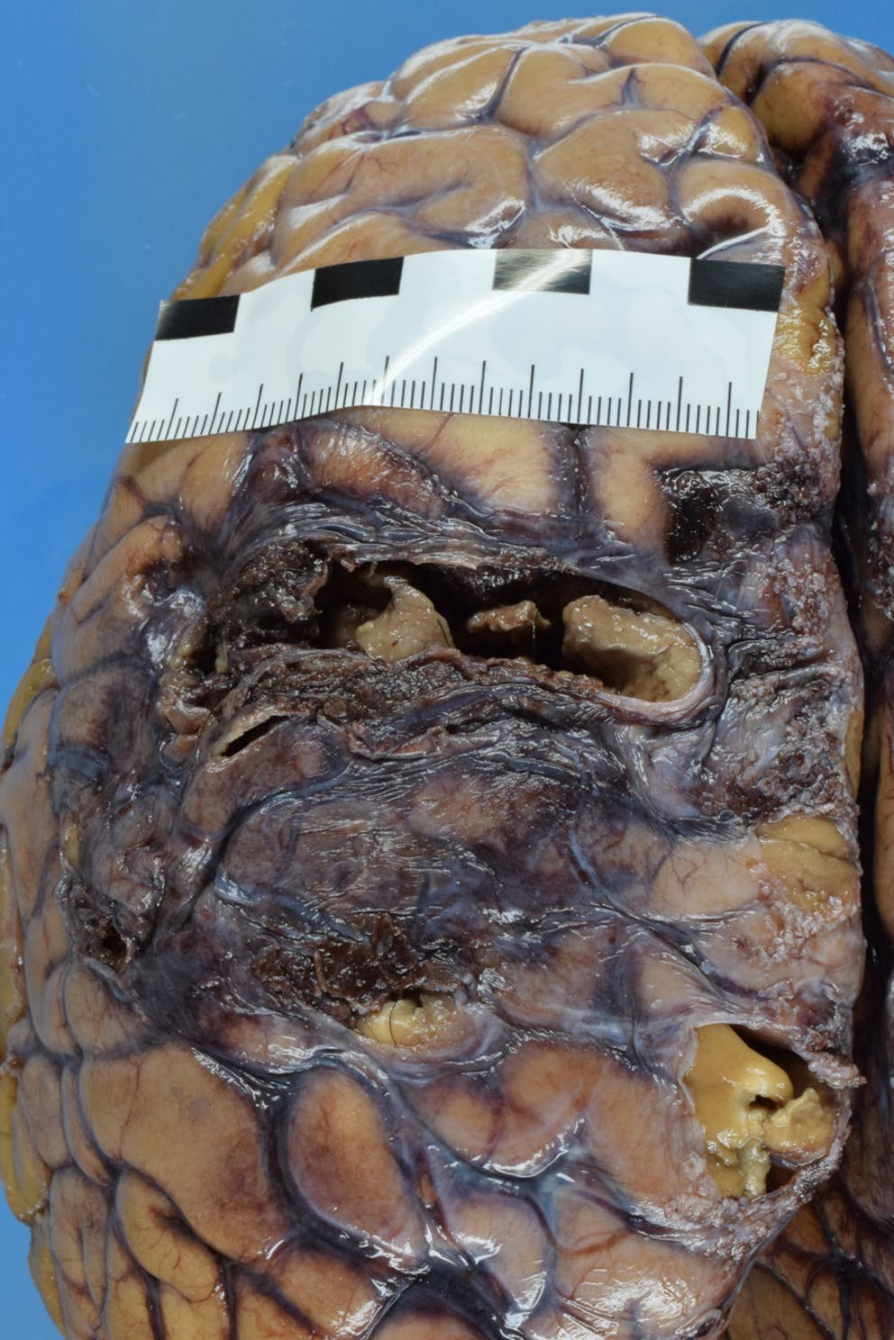
Parenchymal injury in the left cerebral hemisphere after formalin fixation

**Fig. 3b:**
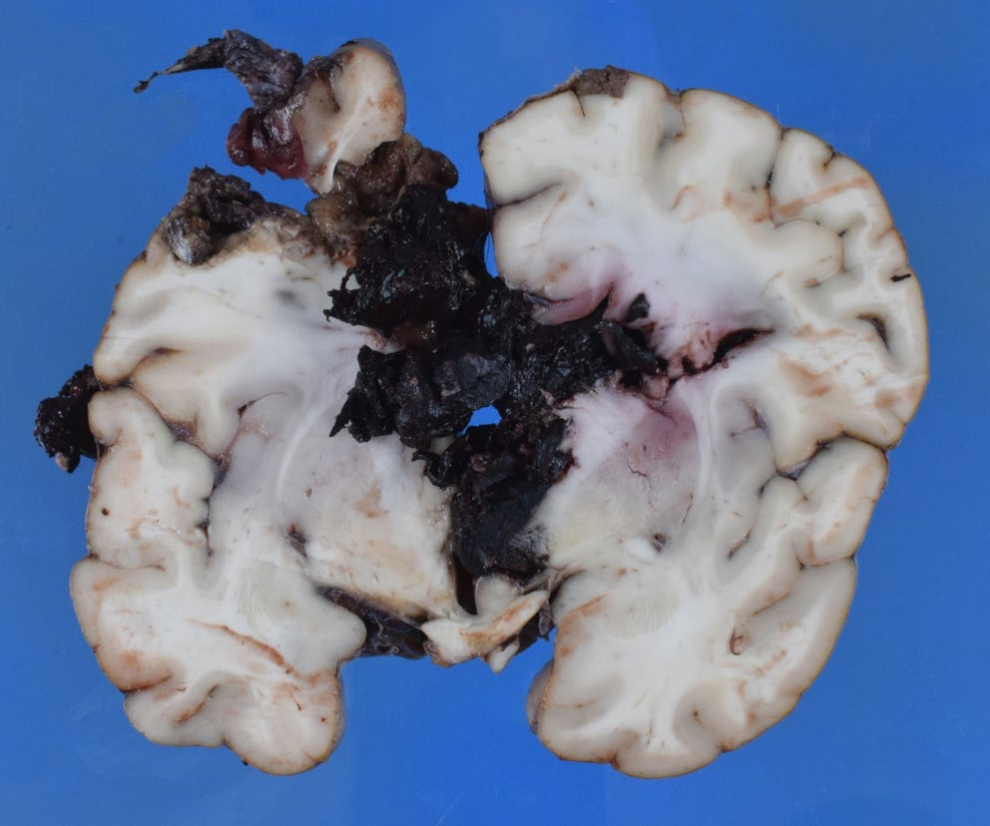
Stab channel with road-like tissue destruction of both hemispheres after formalin fixation

**Fig. 4a:**
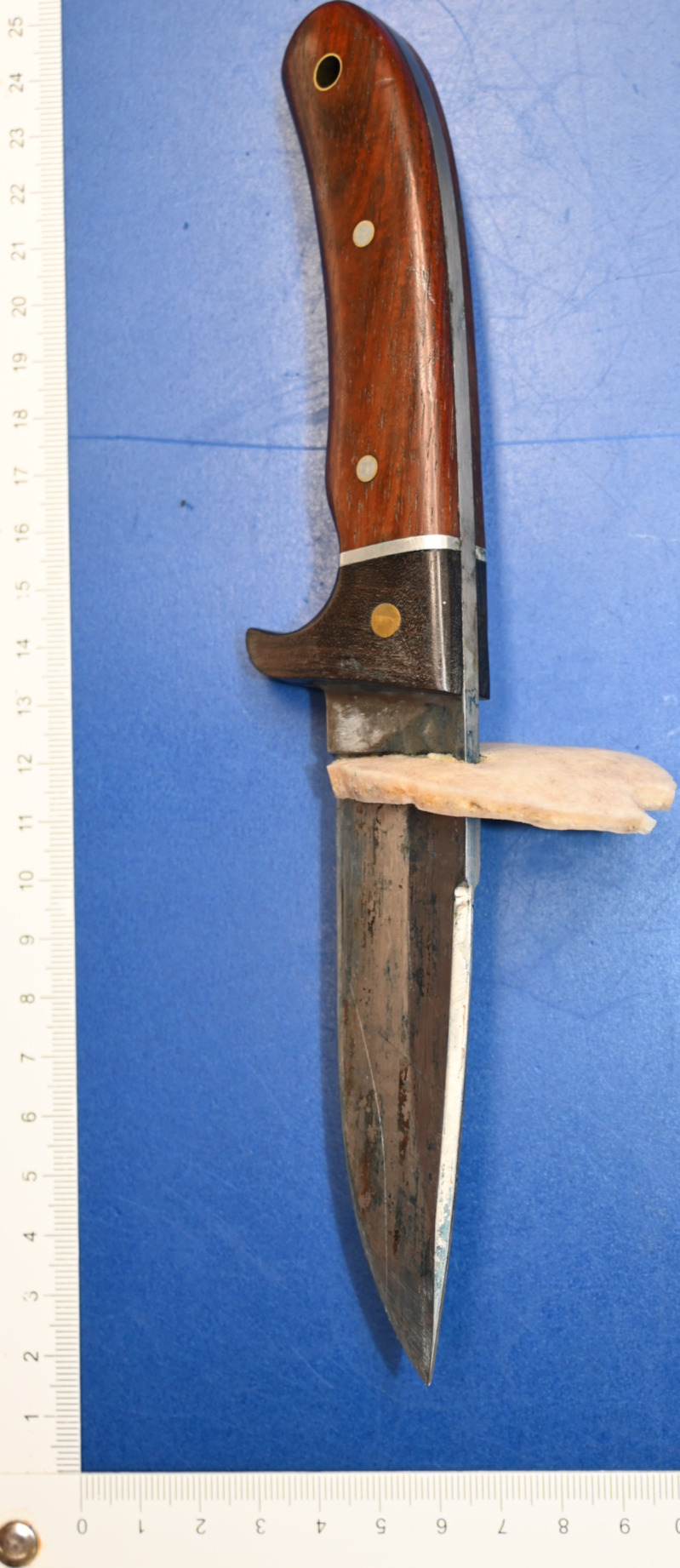
Extensive intracranial stab channel

**Fig. 4b:**
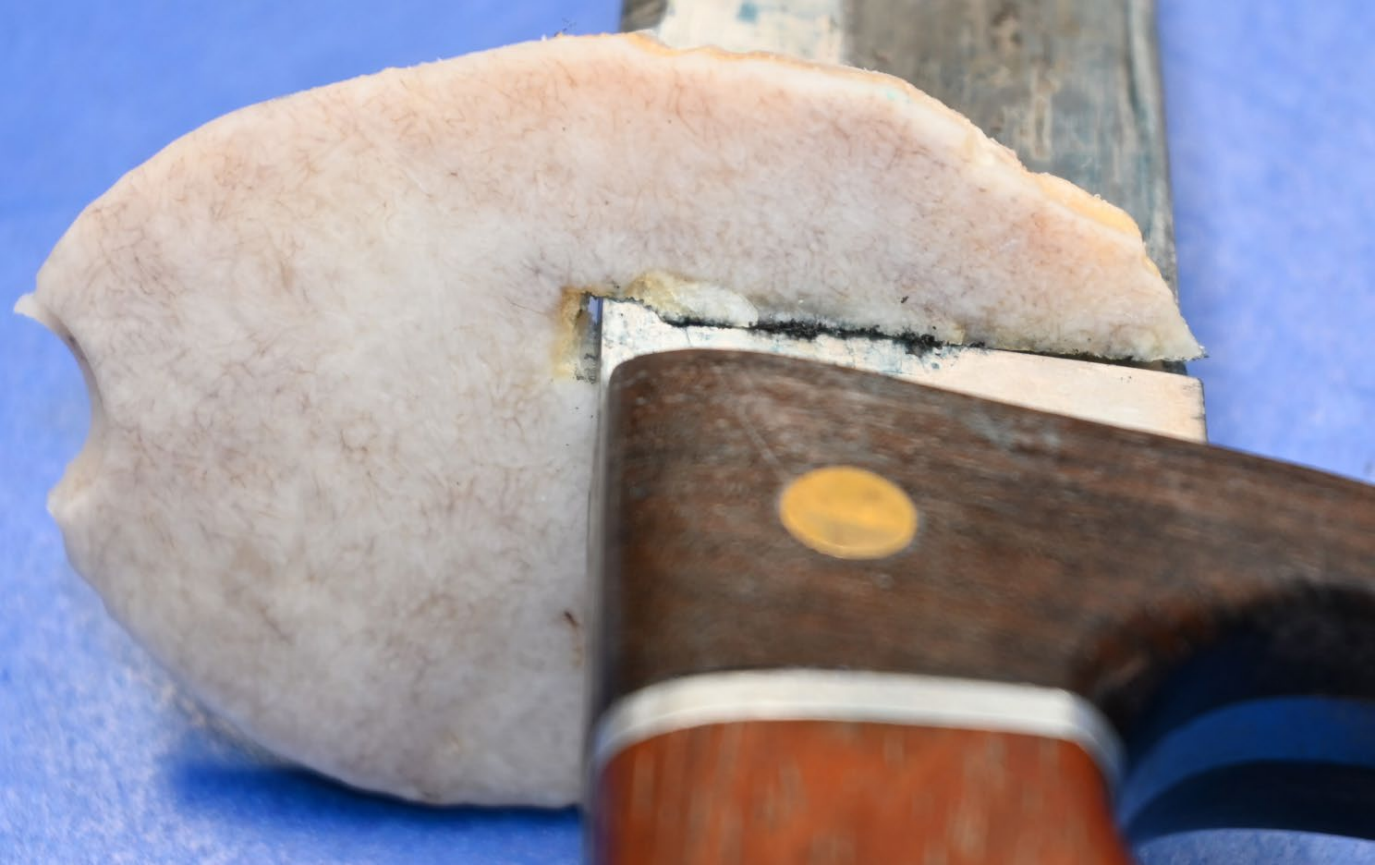
Macerated bone shard with corresponding murder knife match exactly

## Discussion

In sharp force fatalities, the congruence of the stabbing tool and the stab wound on the body surface can usually be assessed at least approximately by comparing corresponding post-mortem photographs with a potential murder weapon. The photographs must then of course meet the requirements of forensic documentation. Corresponding statements are also possible radiologically [1–2]. However, an exact match can usually not be determined without any doubt. The excision of stab wounds to compare stab injury and stab tool is unusual and would be pointless in elastic tissue such as skin. If there are no individual characteristics on the piercing tool or corresponding features on the stab wound, an absolute match with a reliable association between the puncture tool and the puncture itself cannot generally be postulated.

In the case reported here, however, it was possible to unequivocally assign the stabbing tool to the stab wound because the bone shard, which was removed neurosurgically to relieve intracranial pressure, was presented at autopsy, and it exactly matched the instrument of the crime. The length of the intracranial puncture canal could also be reconstructed in this way. A potential murder weapon was found at the scene immediately after the event, but was not available at autopsy due to police investigations. However, at trial about one year later, there was the opportunity to perform a reproducible demonstration of the forensic reconstruction findings *in foro*, even without postmortem radiological findings.

Fatal penetrating skull stabs are an absolute rarity. This applies to accidents, homicides and suicides [1–4]. Accordingly, scientific reports on both the injury morphology and the force required to penetrate a human skull bone are sparse. In addition to skin, fatty tissue and muscles, porcine ribs can also be easily cut by adults using a knife with moderate effort (Energy approx. 1200 N, Force 11–16 J) [5–6]. Bony structures show the greatest resistance to penetrating piercing tools of any human tissue, which is not surprising [7–8]. It was obvious that the stab in the presented case was carried out very powerful, active, intentional and most likely directed downwards. The small fractures on the outside of the skull next to the penetrating bony injury were possibly attributed to the impact of the knife handle but could have been also just the result of the stab without additional blunt force. Although, it was not possible to make reliable statements about the amount of force required, an accident could be excluded.
